# Paresis

**Published:** 1873-04

**Authors:** Andrew K. Minich


*Paresis.* Read before the Northern Medical Association of Philadelphia, September 27, 1872, by ANDREW K. MINICH, M.D.

The term paresis is from the Greek—"I relax," and was introduced by Saloman, a Swede, as a substitute for "general incomplete paralysis of the insane,"—a very unwieldy term, and only partially expressive of the disease under consideration.

Since the days of Calmeil, one of the earliest writers on paresis, it has been recognized as existing in three distinct stages. The symptoms in the first stage are various, and chiefly mental.

There is often a disposition to wander from home, to neglect every-day duties, and to commit petty thefts. Generally there is a change in the morals of the patient; a religious man may become profane, and *vice versa*. Undue importance is attached by the patients to themselves. They imagine they have become wealthy, hold honorable positions, and express determinations to erect churches, colleges, establish new lines of railroads, or project other schemes of great magnitude. The voice in this stage is changed to a slight stutter or stammer.

The more marked symptoms in the second stage are disturbed locomotion and difficult articulation. The walk in this stage is peculiar. Dr. Sankey has well observed "that there is in health a kind of expression in a person's gait from which we may almost divine what kind of errand or business a man is upon." A man walking for exercise has a gait differing from one walking to attend to business. The two chums, with arms hooked, as they saunter along, contrast in a marked manner with the excited broker on Wall street. The gait of a person with paresis is that of a man walking "without a definite object;" and later in the disease walking becomes more a "matter of business." The feet are raised but slightly, are widely separated, and fall flat upon the floor. His arms are to him what the balancing-rod is to the tight-rope walker—a means of maintaining his equilibrium. All the muscles seem affected. Any act requiring muscular interference is attended by phenomena due to a want of harmony in their action. To compare the movements and actions of a paretic to a drunken man would not be amiss, save that the paretic is cautious and deliberate, the drunkard reckless and garrulous. Later in this stage, walking is very difficult, and, from the labor spent upon its performance, a looker-on might well surmise that the performer was making it a specialty. The articulation is drawling and difficult, often the syllables are run into each other, and mumbling takes the place of articulate sounds. Twitching of the muscles around the mouth is common. The tongue when protruded is very tremulous, and is quickly drawn back into the mouth, in spite of urgent requests to the contrary. Grinding of the teeth is frequently indulged in,—a most unpleasant symptom to those within hearing distance. It grates sufficiently hard upon the ear to have such a symptom show itself in a bedridden patient, but to see and hear a man, with a confused stare, with reason dethroned, wandering around grinding his teeth simultaneously with every short step he takes, is almost beyond endurance. The intellectual, moral and motor faculties are all more or less influenced. The senses of touch and taste are impaired to a more marked degree. If a paretic is asked concerning his health, the reply generally will be, "first-rate," "bully," "beautiful," or some other brief expression. I would lay special stress upon the first of these answers, for almost invariably he will be "first-rate." The conversation of the paretic in this stage, as well as in the first, is not disconnected. Fixed delusion is very rare. His ideas bear the impress of change; they are not fixed like those of the monomaniac; *now* he he may be worth a hundred thousand dollars, and cashier of some bank, and one hour hence be a millionaire and chief executive of the nation. He grows *richer* but never *poorer*. "Excelsior" seems to be his motto. The symptoms, then, in this stage are, a want of power to control perfectly the motor, intellectual and moral faculties.

The third stage manifests its advent by dementia, and inability to stand upright or to execute any movement requiring the use of more than a few muscles. This muscular feebleness is more marked in the morning. In this stage the utterance is thick, and often unintelligible. The muscles are constantly twitching. The knees of the patient, when sitting, shake like those of a man suffering from a chill. His teeth chatter, he looks cold, although he tells you that he is "first-rate." Urine and fæces are passed apparently unconsciously. The urine in all the cases that I have observed was extremely phosphatic, not alone in this stage, but also in the second, although somewhat less in the latter. The temperature, according to Mr. Dodge, resident student at the Philadelphia Hospital, who carefully observed it at my request, was found to vary from one-half to one degree, according to the time of day. In the morning it was, in confirmed cases, one hundred to one hundred and five-tenths degrees. In the evening the mercury invariably rose to one hundred and one degrees. The appetite is good, almost voracious, up to the time of dissolution. This, no doubt, assists in prolonging the life of the patient when to the observer death seems imminent. I saw poor John Ambrose, a fine minstrel, whose voice once charmed enthusiastic audiences, literally, like Herod of old, eaten by worms, numbers of which, thick and fat, crawled from his anus and large putrefying burrowing sores. He was a mass of rottenness when death entered his chamber. He partook of a hearty breakfast but a few hours beforehand.

Picking at the bed clothes, and tearing them into shreds with hands and teeth, afford hours of employment to the bedridden paretic. Irritation applied to the soles of the feet produces immediate reflex action. Epilepsy and epileptiform convulsions often occur. Sometimes the patient is seized by apoplexy : indeed, not a few meet their death in this way.

Bed-sores are prone to occur in this stage, their formation being due to the patient's continued supine position, and to the frequent presence of irritating matter. The symptoms then characteristic of paresis are, mental derangement, difficult articulation, and disturbed locomotion.

The cause of this strange malady is still wrapped in mystery. Excess in some form upon the part of the patient, at some period of life, no doubt, lies at the bottom of this disease, as of so many others. The histories of cases confirm this belief. Medical science cannot, however, explain why it is that one person using alcoholic drinks as a beverage will, as a consequence, die of a cirrhosed liver, and another of a cirrhosed brain.

The diagnosis of paresis is not difficult. It may, however, be confounded with muscular atrophy, lead and mercurial palsy, chronic alcoholism, and certain cases of spinal paralysis. It differs from general muscular atrophy in this, that the latter is free from mental derangement. Symptoms of mental derangement, however, might occur in a case of muscular atrophy. In such a case the peculiar form of insanity in paresis, and the condition of the muscles, would serve to distinguish the two affections. Lead and mercurial palsy are less likely to be confounded with paresis. Lead palsy manifests itself more locally. The imperfect locomotion is more circumscribed. The wrists are chiefly affected, and the muscles of the affected parts become wasted. The condition of the gums and the absence of delirium in lead palsy would also serve as distinctive features. Mercurial palsy can also very readily be distinguished. The peculiar trembling of the hand does not occur in paresis, neither is there mental derangement in mercurial palsy. Chronic alcoholism, the result of an abuse of alcoholic drinks, in which the motor and mental functions are affected, is more likely to be mistaken for this disease by a casual observer. This, however, is only true of decided cases of a chronic nature. They present distinctive characteristics. According to M. Thomeuf, the form of mental symptoms usually presented in alcoholism "is a kind of melancholia, with exaggerated fears, frights, delusions of being followed or watched, or of being accused of guilt. The paretic is never afraid, is not melancholy, generally entertains grand ideas, and is never unwell." All those that came under our own observation confirm this statement of Thomeuf. All would express themselves as being "first-rate," and this in tones, to use the words of Dr. Bucknill, "in which could be detected the signs of incurable disease." The appetite in alcoholism is deficient; that of the paretic can scarcely be appeased. Epileptic and epileptiform convulsions occur in paresis; delirium tremens in alcoholism. The latter is curable; the paretic always dies. Certain cases of spinal disease in which the paralysis is more or less general, may be complicated with delirium, and thus be mistaken for paresis. The history of the case, the condition of the spinal cord, and the nature of the delirium, would serve to point out the true diagnosis.

The pathology of this strange malady has been a fruitful source of discussion in later times. Observers do not agree as to its nosological position. Some view it as a form of mania upon which are engrafted paretic symptoms. This view is refuted by the fact that symptoms of paresis never occur in cases of chronic mania. Others maintain that paresis may exist without paralytic manifestations, and recognize it by the name of "ambitious mania." Ambitious mania, though it resembles paresis in the character of its delusions, is a separate affection. Paresis is ambitious mania, but it is something more. We have seen cases of chronic mania, of simple insanity, without any apparent physical disturbance, that even now pace the wards and strut along the corridors of the Insane Department of Blockley Hospital, whose imagination knows no limit. One sports with millions, another is the Saviour of the world, while several proudly march along, conscious that the world recognizes them as ruling monarchs. The majority of observers, however, look upon paresis as a separate affection—a disease *per se.*

It presents characteristics similar to other affections. It is influenced by locality. Dr. Grey makes mention of the fact that in the Edinburgh Asylum there are usually to be found from twenty to twenty-five cases, while Montrose Asylum, about twentyfive miles distant, and containing about three hundred patients, has none. The disease is more prevalent in the eastern and northern parts of America than in the southern and western. Males are much more subject to paresis than females. Statistics prove the proportion to be about seven of the former to one of the latter. Age exerts an influence. Most patients are between the ages of thirty and forty years. Cases occur occasionally, however, to prove that neither the young nor aged are wholly exempt. All paretics are insane. Insanity is one of the principal symptoms. This mental derangement is manifested by an exalted imagination, —very rarely presenting itself in a different form. Respecting the pathology of paresis, some view it as an inflammation. M. Parchappe says, "all facts afforded by pathological research agree in confirming the inflammatory nature of the characteristic lesion of the cortical substance of the brain." M. Belhomme expresses a belief that "paresis is an encephalitis of a peculiar kind."

The post-mortem appearances are not constant in every respect. Sometimes there is softening, sometimes the brain is abnormally firm. This difference is probably due to the comparative duration of the disease. The arachnoid and pia mater generally present traces of inflammatory change. The microscope has detected in most cases a varicose condition of the capillary vessels of the cortical substance, and also a marked increase of its connective tissue. This increase of the connective tissue of the cortical substance is the most constant of the morbid conditions. It is, pathologically speaking, a cirrhosis of the cortical portion of the brain, the result of a slow inflammation. There is no doubt that the disease is cerebral. The impaired locomotion is not owing to muscular paralysis, but to something interfering with co-ordinate action. The constantly cheerful answer which the patient ever gives in regard to his or her health is due to one of two conditions, or to both. It may be but the result of a feeble conception. The brain in its diseased condition, with its cells compressed by the superabundant connective tissue, may be incapable of taking cognizance of the body's condition. The fault does not seem to be in the afferent or efferent nerves. They faithfully transmit impressions, as is shown by the immediate reflex movements that take place in response to any irritation applied to the surface of the body. It may be that even though the patient's brain is capable of appreciating pain and poverty with their accompanying shadows, yet his peculiar form of insanity, his ambitious mania, his radiant line of thought, will scatter those shadows as the wind scatters the leaves of autumn. His judgment has fled, and as his idea of wealth and splendor is not corrected by his bed of straw and lonely cell, in like manner his idea of personal comfort and perfect health may not be corrected by painful sores and fatal disease.

The prognosis is always unfavorable. A few cases of recovery are reported. It may be that the diagnosis was not correct. Recovery, it is stated, was preceded by a general breaking out of boils.

Of the treatment very little can be said. Aside from attention to the general comfort and cleanliness of the patient, it consists merely of a "meditation on death;" of reading from time to time the inscriptions upon the mile-stones as our buoyant patient totters along the downward road to meet his inevitable doom. He is an inmate of that ward above the entrance of which is written, "For Incurables,"—a department which, through the deeply-searching spirit of modern science, may ere long, we hope, be made vacant, and the unfortunate paretic among the rest be restored to health, and ushered from that abode of fate.—*Phil. Med. Times.*

